# Patterns of self-reported alcohol and drug use among children and youth: Mozambique violence against children survey (VACS) 2019

**DOI:** 10.1186/s12889-025-22360-9

**Published:** 2025-03-27

**Authors:** Cynthia Semá Baltazar, Auria Ribeiro Banze, Rachid Muleia

**Affiliations:** Instituto Nacional de Saúde, P.O. Box 264, Maputo, Mozambique

**Keywords:** Substance use, Adolescents, Youth, Mozambique, Violence against children survey (VACS)

## Abstract

**Background:**

Substance use among youth has significant implications for health, development, and society. In Mozambique, where youth form a large segment of the population, understanding the prevalence and factors associated with alcohol and drug use is essential for effective public health strategies. This study aimed to assess the prevalence and factors associated with alcohol and drug use among adolescents and young adults in Mozambique.

**Methods:**

This study is a secondary data analysis of the 2019 Violence Against Children Survey (VACS), a nationally representative cross-sectional survey targeting individuals aged 13–24 years. Data collection were collected using face-to-face interviews with a structured questionnaire. The analysis focused on the prevalence and patterns of self-reported alcohol and drug use and identified potential socio demographic and behavioral factors associated with substance use among the youth.

**Results:**

The overall prevalence of alcohol use was 29.7%, and drug use was 22.5%. Among individuals aged 18–24 years, alcohol use was significantly higher (aOR = 3.8, 95% CI: 2.9–4.9, *p* < 0.001) compared to those aged 13–17 years, while drug use followed a similar pattern (aOR = 2.4, 95% CI: 1.6–3.5, *p* < 0.001). Gender differences were observed, with females being significantly less likely to report drug use (aOR = 0.5, *p* < 0.001). Marital status was associated with a lower likelihood of alcohol use, as married or cohabiting individuals reported less alcohol use (aOR = 0.6, *p* < 0.001). Higher educational attainment was associated with an increased likelihood of alcohol use (aOR = 1.8, *p* < 0.001). Employment in the past 12 months was protective against both alcohol (aOR = 0.7, *p* < 0.001) and drug use (aOR = 0.7, *p* = 0.014). Notably, those who experienced sexual violence in childhood were less likely to consume alcohol (aOR = 0.4, *p* < 0.001) and use drugs (aOR = 0.5, *p* = 0.004), while those whose first sexual intercourse was pressured or forced were more likely to engage in substance use.

**Conclusion:**

The findings highlight the significant prevalence of alcohol and drug use among adolescents and young adults in Mozambique, with notable socio-demographic and behavioral disparities. Factors such as age, gender, marital status, educational attainment, and employment status influence substance use patterns. Additionally, traumatic experiences, including childhood sexual violence and forced or pressured first sexual intercourse, play a complex role in shaping substance use behaviors. These findings emphasize the need for integrated public health interventions that address prevention, mental health support, and socio-economic disparities to reduce substance use and promote the well-being of Mozambique’s youth.

## Background

Drug and alcohol use among children and youth are complex behaviors with profound implications for individual health, developmental trajectories, and society at large. These substances significantly influence cultural norms, peer interactions, and personal experiences. Globally, the early onset of substance use is usually associated with numerous adverse outcomes, such as academic underperformance, mental health disorders, and a propensity towards risky behaviors [1, 2]. People of younger ages are disproportionately affected by substance use compared with people of older ages. The misuse of these substances is a leading cause of morbidity and mortality among young people. The World Health Organization (WHO) reports that over a quarter of individuals aged 15–19 worldwide are current drinkers, totaling 155 million adolescents, with 4.7% having experimented with cannabis.

Youth constitute a significant segment of Mozambique’s population and represent a particularly susceptible group concerning the initiation and experimentation of substance use [3]. This vulnerability is influenced by the country’s socio-political history, including the legacy of colonial rule and prolonged civil conflict, with disrupted social systems and contributed to persistent economic challenges such as high unemployment and income inequality [4]. These factors not only influence the overall health and well-being of children and youth but also affect their risk of substance use. As with many Low-and Middle-Income Countries (LMICs), the regulatory frameworks and prevention programs targeting substance use among young populations are often inadequate, further exacerbating the problem. The prevalence and patterns of self-reported drug, tobacco, and alcohol use among youth and adolescents hold significant ramifications for personal well-being, societal impact, and public health policies [5, 6].

The adverse outcomes associated with early substance use are particularly concerning in settings like Mozambique, where public health systems are under-resourced and the social safety nets are weak [7]. The links between substance use and other high-risk behaviors, including unsafe sexual practices and delinquency, highlight the need for comprehensive approaches to address these interrelated issues. Such insights are critical for informing policies and programs aimed at preventing substance use and supporting the health and development of children and youth [1, 5, 8].

In Mozambique, alcohol consumption among adolescents and young adults is a significant public health concern. Data from the WHO indicate that total alcohol consumption among individuals aged 15–19 is 3.2 L of pure alcohol for females and 5.4 L for males [9]. Additionally, study findings highlight that among drug users, injection drug use is particularly concerning, with a significant proportion of young people reporting injecting drugs as their primary mode of drug consumption [7].

This study aimed to determine the prevalence rates of self-reported drug and alcohol use among youth and adolescents, using the 2019 Mozambique VACS Survey, and to identify and analyze potential socio-demographic, economic, and other risk factors associated with drug, and alcohol use among youth and adolescents.

## Methods

### Data source and study design

This study utilized data collected through the 2019 Mozambique Violence Against Children and Youth Survey (VACS), a nationally representative, cross-sectional household survey aimed at assessing the prevalence and impact of violence against children and youth aged 13–24 years for both males and females. The primary objective of the Mozambique VACS was to measure the prevalence of sexual, physical, and emotional violence against children and youth, and to identify specific risk and protective factors for violence. This data were intended to inform the development and implementation of targeted HIV and violence prevention and response programs and policies in Mozambique [3].

The Mozambique VACS was the first comprehensive, nationally representative survey of its kind in the country and was spearheaded by the Ministry of Gender, Children, and Social Action (MGCAS) with implementation support from ICAP at Columbia University, alongside technical assistance from the United States Centers for Disease Control and Prevention (CDC). Sofala Province, which accounts for approximately 8% of Mozambique’s population, was excluded from the survey due to the significant infrastructure damage and population displacement caused by Cyclones Idai and Kenneth in March and April 2019. Additionally, girls and young woman aged 13–24 were oversampled in Gaza and Zambézia provinces (two geographic areas with high prevalence of HIV that are implementing PEPFAR DREAMS programming) to ensure provincial representative estimates of violence among this group.

The survey used the 2017 Mozambique Population and Housing Census as its sampling frame [10]. Separate samples were drawn for males and females, considering the needed sample size and expected response rates. The survey employed a three-stage cluster sampling design, selecting different Primary Sampling Units (PSUs) for males and females [3].

### Sample size determination

The sample size for the 2019 Mozambique VACS was calculated to ensure adequate statistical power for estimating the prevalence of key indicators, such as substance use, physical violence, and sexual violence, with a margin of error of ± 5% and a 95% confidence level. Separate sample size calculations were performed for males and females to allow for sex-specific analyses. The calculation also accounted for an anticipated response rate of 80% and a design effect of 1.5, to accommodate the multi-stage cluster sampling design. Additionally, oversampling was carried out in specific provinces, such as Gaza and Zambézia provinces, to ensure sufficient representation of girls and youth woman aged 13–24 years in areas implementing PEPFAR DREAMS programming [3].

### Data collection

Data collection was conducted electronically using the Open Data Kit (ODK) software installed on Android tablets. Trained interviewers conducted face-to-face interviews with eligible participants using a structured questionnaire on the tablets [3]. The survey also included voluntary HIV testing. Participants aged 18–24 who had not previously tested positive for HIV and were eligible to receive their results privately, in accordance with national guidelines, were offered an HIV test. The HIV testing procedures followed the WHO’s “Consolidated guidelines on HIV testing services 2015” and Mozambique national guidelines [11].

### Study measures

The VACS utilized a core questionnaire designed for the head of household, alongside separate questionnaires for male and female participants, which were adpated for the Mozambican context. The participant questionnaires covered a comprehensive range of topics, including demographic information, family relationships, education, social connections, gender norms, perceptions of safety, experiences of witnessing violence, sexual history, experiences of violence (physical, sexual, and emotional), violence perpetration, pregnancy, health outcomes, and service use. Participants were asked to report on their experiences of childhood violence as well as violence encountered in the past 12 months.

For childhood violence, estimates were derived from reports by participants aged 18–24, reflecting on experiences before the age of 18. Current violence estimates were based on responses from both 13–17 and 18-24-year-olds, focusing on incidents occurring within the 12 months preceding the survey. This approach facilitated the examination of ongoing patterns and contexts of childhood violence and its connection to HIV in Mozambique.

Sexual violence was broadly defined to include acts such as completed non-consensual sex, attempted non-consensual sex, non-physically pressured sex, and unwanted sexual contact. Participants were asked about four specific types of sexual violence: unwanted sexual touching (including fondling or grabbing without consent), attempted but unsuccessful forced sex, pressured or coerced sex, and physically forced sex.

Physical violence was defined as the intentional use of physical force with the potential to cause harm. Participants were queried about their experiences with physical violence from various perpetrators, including intimate partners (current or previous), peers (such as siblings, schoolmates, neighbors, or strangers), parents or caregivers, and adults in the community (e.g., teachers, police, employers, or community leaders). They were asked whether they had experienced acts such as slapping, pushing, shoving, shaking, having objects thrown at them, being punched, kicked, whipped, beaten with an object, choked, smothered, nearly drowned, intentionally burned, or threatened with or attacked using a weapon.

The present study analyzed two outcomes, namely alcohol consumption and drug use. Alcohol consumption was defined as 1 if the respondent reported to intake alcohol and 0 otherwise. Regarding drug use, the outcome variable was defined as a composite variable, combining two questions, namely: *“Do you currently smoke tobacco on a daily basis*,* less than daily*,* or not at all?”* and *“In the past 30 days*,* have you used drugs such as marijuana*,* pills*,

*ecstasy*,* or sniffed any chemical such as petrol or glue?”*. Therefore, drug use was defined as 1 if the study participant responded yes to either question, and 0 otherwise.

### Statistical analysis

All the statistical analyses were conducted using the R statistical software, version 4.4.1. Descriptive analyses were performed to describe the background characteristics of the respondents and the prevalence of alcohol consumption, and the substance abuse. To explore the factors associated with the outcome variables (alcohol consumption and substance use) we fitted a multivariate logistic regression considering all the factors seen as important associations for the outcome variables. As the number of variables hypothesized to be related to the outcome were not substantial, all the independent variables were included in the multivariate logistic regression without prior variable selection. Both descriptive and multivariate analyses were performed accounting for complex sampling plan employed in the VACS survey, adjusting for sampling weights and clustering. The results are presented in the form of percentages and adjusted odds ratio accompanied by the 95% confidence interval. Statistical significance was assessed at 5% level in the multivariate analysis.

### Ethical considerations

The Mozambique VACS adhered to the World Health Organization’ (WHO) ethical and safety guidelines for conducting studies on violence [12]. The survey protocol received independent review and approval from the Mozambique Ministry of Health’s National Bioethics Committee (CNBS), INS institutional Bioethics Committee for Health (CIBS), and the CDC Institutional Review Board, ensuring the protection of the rights and welfare of all human research participants. The study protocols and the training of interviewers adhered to rigorous standards designed to ensure the safety, privacy, and confidentiality of the children and youth participating in the violence survey.

The informed consent process began by obtaining consent from the head of the household. For participants under 18, parental or guardian consent was required. Participants aged 18 and older, as well as emancipated minors, provided their own written informed consent. Emancipated minors were defined as 13–17 years old who were either married or previously married, living alone without adult supervision (with both parents deceased and not living in a care facility), or those heading a household and caring for siblings.

## Results

### Demographic and behavioral characteristics

A total of 3,008 participants were included in the analysis, comprising 2,129 females (70.8%) and 879 males (29.2%).

Among females, the median age was 18 years (IQR: 15–21), with 42.0% aged 13–17 years. The majority (45.9%) were unmarried, 9.2% had no formal education, and 16.9% had worked in the past 12 months. Regarding experiences of childhood violence, 15.4% reported physical violence, and 21.5% reported sexual violence, with 3.4% subjected to forced sexual acts. In addition, 11.6% reported that their first sexual intercourse was pressured or forced. Sexual risk behaviors were also observed, with 7.9% reporting two or more sexual partners in the past 12 months and 99.2% reporting inconsistent condom use (Table 1).

Among males, the median age was 17 years (IQR: 15–20), with 47.8% aged 13–17 years. The majority (76.4%) were unmarried, and 51.3% had completed secondary or higher education. Experiences of violence during childhood included 19.6% reporting physical violence and 14.5% reporting sexual violence. Among those reporting childhood sexual violence, 2.3% were coerced and 1.9% were physically forced. Sexual risk behaviors were more prevalent among males, with 36.0% reporting two or more sexual partners in the past 12 months and 88.0% reporting inconsistent condom use (Table 1).

Substance use was reported by both sexes, with 29.7% overall prevalence for alcohol use and 22.5% for drug use (data not shown). Alcohol use was more common among males (35.2%) than females (27.1%), while drug use followed a similar pattern (27.1% in males vs. 18.2% in females). Older participants (aged 18–24 years) reported higher prevalence of substance use, with 45.3% for alcohol use and 37.7% for drug use, compared to 8.4% and 6.8%, respectively, among those aged 13–17 years (Table 1).

**Table 1:** Descriptive characteristics of Mozambique participants among 13–24-year-old females and males Mozambique 2019 violence against children

Characteristics	Female (*N* = 2129)		Male (*N* = 879)	
	Weighted %	95% CI	Weighted %	95% CI
**Age of survey participant**				
*Median age (IQR)*	18(15–21)		17(15–20)	
13–17	42.0	38.0–46.0	47.8	42.5–53.1
18–24	58.0	54.0–62.0	52.2	46.9–57.5
Marital status*				
Unmarried	45.9	42.6–49.3	76.4	72.4–80.5
Married or cohabitated	44.9	41.4–48.5	20.4	16.7–24.1
Married in the past	9.2	6.7–11.6	3.2	2.0-4.3
**Highest level of education**				
Never attended school	9.2	7.1–11.4	6.7	4.9–8.6
Primary or less	41.6	38.2–45.0	42.1	37.8–46.3
Completed secondary school or higher	49.2	45.5–52.9	51.3	47.2–55.3
**Employment Status**				
Work in the past 12 month	16.9	13.7–20.0	42.0	37.9–46.2
**Physical and Sexual Violence in Childhood**				
Suffered Physical Violence	15.4	12.3–18.5	19.6	15.4–23.9
Suffered Sexual Violence	21.5	18.5–24.5	14.5	11.3–17.7
**Childhood Sexual Violence**				
Pressured/coerced sex in childhood	4.4	2.9–5.9	2.3	1.1–3.6
Physically forced sex in childhood	3.4	2.1–4.7	1.9	0.8–2.9
Unwanted sexual touching in childhood	8.4	6.0-10.8	4.7	93.7–5.8
**First sexual intercourse was pressured or forced**	11.6	8.8–14.4	4.2	2.1–6.3
**Sexual risk-taking behaviors in the past 12 months**				
Two or more sex partners	7.9	4.8–11.7	36.0	30.1–41.9
Infrequent condom use^a^	99.2	98.3–100.0	88.0	82.2–93.8
**Ever had an STI**	3.7	2.1–5.2	6.3	3.4–8.6
**Substance Use**				
**Alcohol use**	24.4	22.6–26.2	35.2	32.0-38.4
**Drug use**	18.2	16.6–19.8	27.1	24.2–30.0

^*a*^ *Sometimes or never using condoms when having sex with someone in the past 12 months*

### Factors influencing alcohol and drug use among young people

Participants aged 18–24 years old were significantly more likely to use alcohol compared to the younger age group of 13–17 years (aOR = 3.8. *p* < 0.001). Marital status showed an association with alcohol consumption, with married or cohabitating individuals less likely to consume alcohol (aOR = 0.6, *p* < 0.001). Participants with higher educational attainment (completed secondary school or higher) had a significantly increased likelihood of alcohol use (aOR = 1.8, *p* < 0.001) compared to those who never attended school. Conversely, employment within the past 12 months was associated with reduced alcohol use (aOR = 0.7, *p* < 0.001).

Childhood experiences of sexual violence were associated with lower alcohol consumption (aOR = 0.4, *p* < 0.001), while individuals whose first sexual intercourse was pressured or forced were more likely to consume alcohol (aOR = 3.2, *p* < 0.001). (Table 2).

The patterns of drug use also show notable associations with various factors. Like alcohol use, drug use was more prevalent among participants aged 18–24 years compared to those aged 3–17 group (aOR = 2.4, *p* < 0.001). Females were significantly less likely to use drugs compared to males (aOR = 0.5, *p* < 0.001). Employment within the past 12 months was associated with a lower likelihood of drug use (aOR = 0.7, *p* = 0.014).

Participants who experienced unwanted sexual touching in childhood were less likely to use drugs (aOR = 0.4, *p* = 0.016). Similarly, individuals who experienced sexual violence in childhood had a reduced likelihood of drug use (aOR = 0.5, *p* = 0.004). However, those whose first sexual intercourse was pressured or forced were more likely to use drugs (aOR = 2.5, *p* = 0.023). (Table 2).

**Table 2:** Associations between demographic, social, and behavioral factors and drug use among young people in Mozambique

Survey Characteristics	Alcohol Use			Drug Use		
	*n* (%)	aOR (95% CI)	*P*-value	*n* (%)	aOR (95% CI)	*P*-value
**Age of survey participant**						
13–17 years	1333 (91.6)			1334 (90.3)		
18–24 years	1672 (69.3)	3.8 (2.9–4.9)	< 0.001	1672 (67.8)	2.4 (1.6–3.5)	< 0.001
**Sex**						
Male	879 (77.6)			879 (75.7)		
Female	2126 (80.8)	1 (0.8–1.3)	0,804	2129 (79.7)	0.5 (0.3–0.7)	< 0.001
**Marital status**						
Unmarried	1617 (82.0)			1618 (81.3)		
Married or cohabitated	1187 (76.6)	0.6 (0.5–0.7)	< 0.001	1187 (74.3)	0.9 (0.6–1.2)	0,347
Married in the past	197 (66.8)	1 (0.7–1.3)	0,806	197 (63.6)	1.6 (1.0-2.5)	0,063
**Highest level of education**						
Never attended school	248 (86.4)			248 (84.7)		
Primary or less	1354 (85.1)	1.3 (0.9–1.8)	0,170	1355 (83.2)	1.3 (0.8–2.2)	0,348
Completed secondary school or higher	1394 (73.2)	1.8 (1.3–2.6)	< 0.001	1394 (72.3)	1.6 (1.0-2.7)	0,065
**Employment Status**						
Work in the past 12 month	712 (70.7)	0.7 (0.6–0.9)	< 0.001	712 (68.1)	0.7 (0.5–0.9)	0,014
**Physical and Sexual Violence in Childhood**						
Suffered Physical Violence	434 (77.4)	1 (0.8–1.2)	0,850	434 (76.4)	0.7 (0.5-1.0)	0,059
Suffered Sexual Violence	461 (64.4)	0.4 (0.3–0.6)	< 0.001	461 (63.8)	0.5 (0.3–0.8)	0,004
**Childhood Sexual Violence**						
Pressured/coerced sex in childhood	90 (77.1)	0.8 (0.4–1.4)	0,427	90 (75.8)	1.1 (0.5–2.4)	0,891
Physically forced sex in childhood	76 (69.7)	0.8 (0.5–1.2)	0,275	76 (69.7)	0.8 (0.4–1.6)	0,455
Unwanted sexual touching in childhood	156 (66.5)	0.9 (0.6–1.3)	0,462	156 (66.5)	0.4 (0.2–0.9)	0,016
**First sexual intercourse was pressured or forced**	153 (77.4)	3.2 (1.9–5.6)	< 0.001	153 (75.9)	2.5 (1.1–5.3)	0,023
**Sexual risk-taking behaviors in the past 12 months**						
Two or more sex partners	280 (66.0)	0.8 (0.6–1.1)	0,135	280 (63.2)	0.6 (0.4–0.9)	0,011
Infrequent condom use	1600 (72.5)	1.5 (1.0-2.2)	0,067	1600 (70.2)	0.7 (0.3–1.4)	0,275

## Discussion

Our results indicate an association between age and substance use, with individuals aged 18–24 years reporting higher rates of alcohol and drug consumption compared to those aged 13–17. This suggests that older adolescents and young adults in Mozambique may be particulary vulnerable to substance use, highlighting potential implications for public health, social welfare, and economic development. This finding aligns with global trends and may be attributed to factors such as increased autonomy, peer pressure, and exposure to substance-use environments prevalent among this age group [13–16]. The consequences of early substance use can be far-reaching, including impaired cognitive function, academic difficulties, increased risk of accidents, and involvement in crime [1, 2, 13].

Gender differences in substance use were also evident, with females being significantly less likely to use alcohol and drugs compared to males. This finding aligns with existing literature that suggests gender norms and societal expectations may play a role in discouraging drug use among females [17, 18]. However, this also highligths the need for gender-sensitive approaches in substance use prevention programs, ensuring that both males and females receive appropriate education and support [19, 20].

In our findings, marital status emerged as a protective factor against alcohol use, with those who are married or living as married showing a decreased likelihood of alcohol consumption. This may be related to the stabilizing influence of marriage and the associated social responsibilities that can deter risky behaviors like excessive alcohol use [21]. In contrast, individuals with higher educational attainment were more likely to consume alcohol. This could be reflective of lifestyle changes associated with higher education, such as increased social interactions and increased exposure to environments where alcohol use is more prevalent [15, 22, 23].These findings underscore the importance of integrating responsible drinking education into academic and professional settings to mitigate potential health risks associated with alcohol consumption.

Employment was found to have a protective effect against both alcohol and drug use among youth and adolescent, with those employed in the past 12 months being less likely to engage in these behaviors [22]. Employment likely provides structure, purpose, and financial stability, which can reduce the appeal or necessity of engaging in substance use as a coping mechanism. This highlights the importance of economic opportunities and employment programs in reducing substance use among young people in the country [24, 25].

The findings that individuals who suffered sexual violence during childhood are less likely to consume alcohol and use drugs highlight a complex and potentially counterintuitive relationship between traumatic experiences and substance use. While it might be expected that traumatic experiences such as sexual violence would lead to higher rates of substance use as a coping mechanism [26, 27], the data suggests otherwise in this context. This could indicate that these individuals may employ alternative coping mechanisms, possibly avoiding substances to retain a sense of control or due to heightened vulnerability and awareness of risks associated with substance use. Additionally, societal factors, such as stigma and the support systems available, may also play a role in shaping these behaviors [26, 28, 29].

Interestingly, the data also shows that individuals whose first sexual intercourse was pressured or forced are more likely to engage in both alcohol (aOR = 3.2, *p* < 0.001) and drug use (aOR = 2.5, *p* = 0.023). This suggests that early traumatic sexual experiences increase vulnerability to substance use, possibly as a means of coping with the emotional and psychological aftermath of such experiences. The increased likelihood of substance use in this group could also be linked to ongoing trauma, mental health challenges, or social isolation that often accompanies such experiences. This aligns with existing literature that shows a strong association between early adverse experiences, such as sexual violence, and higher rates of substance use disorders later in life [26, 27, 29]. These findings underscore the importance of targeted interventions that address both the prevention of sexual violence and the provision of mental health support for survivors, to mitigate the risk of subsequent substance use and its associated harms.

Overall, these findings emphasize the complex interplay of age, gender, marital status, education, and employment in influencing substance use among youth in Mozambique. They point to the need for multifaceted public health strategies that consider these factors in designing effective prevention and intervention programs. Addressing substance use in this context requires a nuanced understanding of the socio-cultural and economic landscape of Mozambique, ensuring that interventions are both culturally relevant and targeted to the specific needs of different demographic groups.

## Conclusion

Our study underscores the complex interplay of factors contributing to substance use among Mozambican youth. Our findings illuminate the heightened vulnerability of older adolescents and young adults, particularly males, to alcohol and drug use. While employment and marital status appeared to offer some protection, the pervasive impact of childhood sexual violence on substance use trajectories requires attention.

These findings emphasize the need for targeted public health interventions that address the specific needs of different demographic groups. Programs aimed at preventing substance use should consider the influence of education, employment, and traumatic experiences. Additionally, there is a critical need for mental health support for survivors of sexual violence to mitigate the risk of subsequent substance use.

By understanding and addressing the underlying factors contributing to substance use, policymakers and public health practitioners can develop more effective strategies to support the health and well-being of Mozambique’s youth.

## Data Availability

All study information and datasets are available through the Mozambique National Institute of Health (INS) data repository for researchers who meet the criteria for access to confidential data. Data from the VACS study can be accessed by contacting the authors via the INS website: www.ins.gov.mz.
